# Automatic road damage recognition based on improved YOLOv11 with multi-scale feature extraction and fusion attention mechanism

**DOI:** 10.1371/journal.pone.0327387

**Published:** 2025-09-26

**Authors:** Linxuan Zhang, Yu Deng, Yuelin Zou

**Affiliations:** 1 School of Civil Engineering and Architecture, Guangxi University of Science and Technology, Liuzhou, China; 2 State Grid Xinjiang Information & Telecommunication Company, Urumqi, China; University of Education, PAKISTAN

## Abstract

Rapid urbanization and growing traffic volumes have increased the demand for efficient and accurate road damage detection to ensure traffic safety and optimize maintenance. Traditional manual and vehicle-mounted inspection methods are often inefficient, costly, and prone to error. Deep learning-based approaches have made progress but still face challenges in detecting small objects, handling complex backgrounds, and meeting real-time requirements due to high computational costs and limited generalization. This study proposes an improved road damage detection method based on YOLOv11, incorporating a Tiny Object Detection Layer for enhanced small object recognition through high-resolution and multi-scale feature fusion. A Global Attention Mechanism is integrated to emphasize critical regions and suppress background noise. Additionally, lightweight convolution modules (C3k2CrossConv and C3k2Ghost) optimize the network to reduce computational complexity and improve inference speed. Experimental results on the RDD2022 dataset show that the YOLOv11-ATL model achieves 3.2% and 3.1% gains in mAP@0.50 and mAP@0.50:0.95, respectively, demonstrating robust performance in complex environments while maintaining a favorable balance between accuracy and efficiency. Overall, the proposed approach offers a practical and effective solution for intelligent road damage detection, supporting urban infrastructure management and intelligent transportation systems.

## 1 Introduction

With the rapid pace of urbanization and increasing traffic volumes, maintaining and restoring road quality has become a critical priority for governments and municipal authorities. Good road conditions are fundamental to ensuring travel safety, improving transportation efficiency, and supporting economic development and social stability. Road damage including cracks, potholes, spalling, and settlement negatively impacts not only driving comfortand road aesthetics but also the structural integrity and load-bearing capacity of pavements, ultimately threatening both infrastructure longevity and user safety [1]. Early detection, diagnosis, and timely repair of such damages are therefore essential to prevent further deterioration, avoid accidents, extend service life, and reduce maintenance costs.

Traditional road damage detection methods primarily rely on manual inspections and vehicle-mounted monitoring systems, which suffer from low efficiency, high operational costs, and susceptibility to human error. In contrast, recent advances in artificial intelligence (AI), particularly deep learning, have enabled automated and intelligent detection approaches that greatly minimize human intervention and improve detection accuracy. Consequently, automated road damage recognition has become an indispensable component of modern intelligent transportation systems (ITS) [2], autonomous driving technologies, and smart city infrastructures.

Driven by rapid developments in computer vision and deep learning, numerous methods have been proposed for automated road damage detection. Popular deep learning architectures such as YOLO, Mask R-CNN [3], Faster R-CNN [4], U-Net [5], and convolutional neural networks (CNNs) have demonstrated strong capabilities in image classification, localization, and segmentation tasks. CNNs excel at extracting discriminative features from damaged road images, supporting accurate classification or regression of damage types. However, they can struggle with detecting small targets and complex backgrounds. YOLO algorithms provide real-time object detection through grid-based bounding box and class prediction, but may be less effective for small or overlapping objects. Faster R-CNN offers high accuracy via region proposal networks but is computationally intensive, limiting real-time application. U-Net achieves precise pixel-level segmentation through encoder-decoder architectures but requires substantial computational resources and may suffer in cases of ambiguous boundaries. Mask R-CNN extends Faster R-CNN by adding instance segmentation masks, which enhances performance in complex scenes at the cost of higher annotation and computation demands.

Although deep learning has brought considerable improvements to road damage detection, several challenges remain. The models often require heavy computation, struggle to detect small or subtle damage, don’t perform consistently well in complex environments, and sometimes fail to generalize across different real-world situations. To tackle these issues, we enhanced YOLOv11 [6] and developed a new model called YOLO-ATL. This model combines a global attention mechanism with multi-scale feature fusion, enabling it to better capture important details and significantly improve the accuracy and robustness for detecting small and complex damages. Moreover, by using lightweight convolutional modules, YOLO-ATL reduces computational costs while maintaining strong performance. This balanced design addresses many common challenges and provides an efficient, reliable solution for automated road damage detection, suitable for practical applications in smart transportation and infrastructure monitoring.

The principal contributions of this study are summarized as follows:

- An enhanced detection model, YOLO-ATL, is proposed, which is built upon the YOLOv11 framework. The model achieves improved detection accuracy and robustness while maintaining a balanced between parameters and computational overhead.
- A comparative analysis of several prominent attention mechanisms is conducted to evaluate their impact on model performance and complexity. Based on this evaluation, the GAM is identified as the most effective and subsequently integrated into the backbone network of YOLOv11, resulting in enhanced focus on critical features and improved feature extraction capabilities.
- A novel integration of a dedicated tiny object detection layer combined with multi-scale feature fusion is introduced into the neck network of YOLOv11. This integration substantially improves the model’s performance in detecting small objects.
- The original C3k2 module within the backbone network is replaced with a lightweight C3k2CrossConv module, leading to reductions in both model parameters and computational complexity. The effectiveness of these architectural modifications is validated through extensive experimental evaluations.

## 2 Related work

### 2.1 Detection methods for road damage

The accelerated progress in deep learning techniques has substantially propelled advancements in road damage detection methodologies, thereby addressing the inherent limitations associated with manual inspection and traditional image processing approaches, particularly with respect to efficiency, accuracy, and subjectivity. These advances address the inherent drawbacks of manual inspection and conventional image processing methods, including low efficiency, limited accuracy, and strong subjectivity. In recent years, The rapid advancement of deep learning technology has greatly facilitated technological innovations in the field of object detection. Presently, target detection approaches can be classified into two categories: two-stage models relying on region proposals and one-stage models employing direct regression of bounding boxes. Two-stage models typically involve two steps: first, candidate regions are generated through feature extraction to cover possible target locations; then, these regions are passed into a classification network for further feature analysis and to determine the presence and category of targets. Representative two-stage object detection algorithms include Mask R-CNN, Faster R-CNN, R-FCN [7] and Fast R-CNN [8]. These approaches generally operate by first generating candidate regions of interest, followed by a refined detection step to achieve higher accuracy. Although two-stage detection methods have achieved notable accuracy, their intricate network designs and relatively low inference speeds restrict their deployment in real-time applications. Conversely, one-stage detection models bypass the candidate region proposal phase and employ regression techniques to directly predict object classes and bounding boxes, thereby substantially enhancing detection speed. Typical examples include RetinaNet [10], SSD [9], and the YOLO series [6,11–18]. Particularly, the YOLO algorithm has emerged as a widely adopted mainstream method in real-time object detection due to its combination of high processing speed and comparatively high detection accuracy. With continuous iteration from YOLOv1 to the latest YOLOv11, the model’s has achieved significant advancements in detection performance and processing speed, thereby facilitating technological innovation and broadening practical applications within the field. In the domain of road damage detection, more researchers have begun applying these object detection techniques. For instance, NaddafSH et al. [19] employed the EfficientDet-D7 [20] model for asphalt road damage recognition and achieved commendable results in the 2020 IEEE Big Data Challenge. Despite its high accuracy, the model’s heavy structure and slow response time make it less suitable for real-time use. Additionally, Seungbo Shim et al. [21] proposed a method that integrates GAN-based generation with semi-supervised training strategies, reaching an average recognition accuracy of 81.54%. Hacıefendioğlu et al. [22] used Faster R-CNN to investigate how factors such as shooting height, distance, weather, and illumination affect concrete road damage detection, providing valuable insights for practical scenarios. Moreover, Arya et al. [23] utilized a lightweight architecture for road damage detection across a multi-country dataset, demonstrating the model’s effectiveness; nevertheless, its accuracy and adaptability in complex environments could still be enhanced. Pei et al. [24] combined Cascade R-CNN with multiple data augmentation techniques to enhance detection accuracy, achieving strong results in the 2020 Global Road Damage Detection Challenge. However, the high computational cost of Cascade R-CNN still poses challenges for real-time deployment. Recently, Wang Xueqiu et al.[25] introduced the BL-YOLOv8 model, which demonstrated strong performance by attaining an F1 score of 0.87 on the RDD2022 dataset[26], demonstrating that YOLOv8 effectively combines real-time performance with high accuracy and suitability for mobile deployment Overall, although existing methods have made significant strides in road damage detection, there remains considerable scope for improving detection accuracy, accelerating processing speed, and enhancing robustness in complex environments.

### 2.2 YOLOv11 network architecture

**Fig 1:**
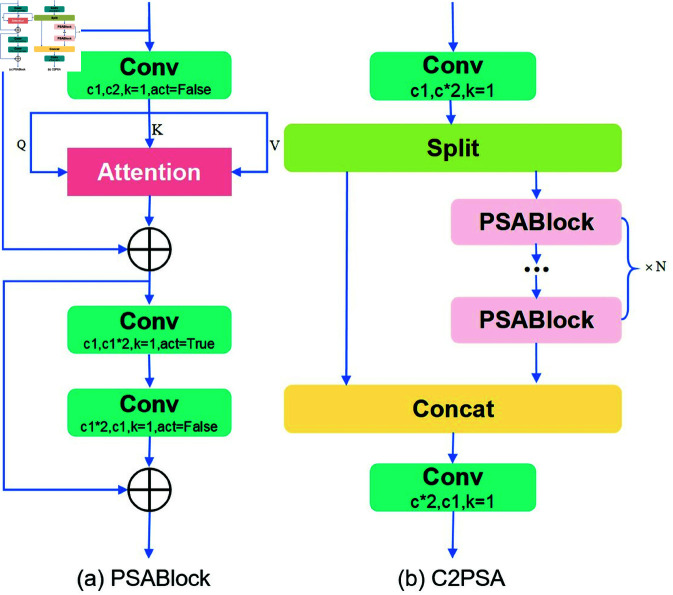
The network architecture of the C2PSA module.

YOLOv11 builds upon previous versions with significant improvements in feature extraction, fusion, and detection performance. Its core architecture consists of an input module, backbone network, neck module, and output detection head. The input module preserves the image aspect ratio and applies adaptive grayscale padding to ensure proper resizing for various detection tasks without distortion or information loss. The backbone network employs multiple stacked convolutional layers along with an enhanced C3k2 module, which offers more efficient and higher-quality multi-scale feature extraction compared to YOLOv8. To strengthen multi-scale feature fusion, YOLOv11 incorporates the SPPF module, which uses multi-scale pooling to enrich feature representation, making the network more adaptable to complex scenes. Additionally, the C2PSA module—an upgraded version of the traditional C2f integrates a pointwise spatial attention mechanism to better focus on critical regions while suppressing irrelevant information, and supports selective residual connections to optimize gradient flow. The architecture of the C2PSA module is illustrated in Fig 1. The neck module builds on YOLOv8’s FPN and PAN structures, combining upsampling and downsampling to fuse semantic and localization information, with two added depthwise separable convolution layers further enhancing classification efficiency and detection accuracy. The output detection head continues the one2one design from YOLOv10, assigning a unique prediction to each ground truth object, thereby eliminating the need for Non-Maximum Suppression and simplifying inference while improving speed and efficiency. The detection head consists of two branches: one2many, which uses multi-label assignment to provide rich supervisory signals, and one2one, which precisely locates and classifies targets; together, they enhance detection performance. The position regression branch utilizes two standard convolutional layers for deep feature fusion, followed by a single convolutional layer to predict spatial coordinates. Meanwhile, the classification branch integrates depthwise separable convolution with pointwise convolution to enhance inter-channel communication, ultimately producing class probability distributions through a convolutional layer activated by Softmax. This architecture ensures a balance between detection accuracy and computational efficiency. Overall, YOLOv11 achieves a substantial enhancement in model expressiveness and robustness while maintaining high efficiency.

**Fig 2:**
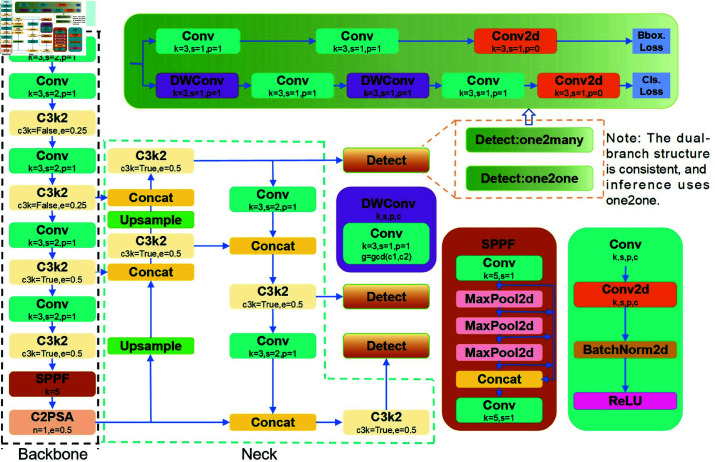
The network architecture of the YOLOv11 model.

The overall architecture of YOLOv11 is illustrated in Fig 2.

### 2.3 Attention mechanisms

Attention mechanisms, inspired by the human cognitive process of selective focus, have become essential components in deep learning architectures. They dynamically enhance relevant features while suppressing irrelevant information, significantly improving performance across tasks. In image analysis, Attention mechanisms are commonly classified as channel attention and spatial attention: channel attention assigns different weights to feature channels based on their relevance, while spatial attention emphasizes the spatial location information. SENet [27] is a seminal work in channel attention. It uses fully connected layers and global average pooling to automatically adjust the importance of each feature channel. To reduce computational complexity, ECA [28] replaces the fully connected layers with one-dimensional convolutions, achieving an effective balance between efficiency and performance. For spatial attention, CBAM [29] sequentially combines channel and spatial attention to help models identify both “what” and “where” to focus. Coordinate Attention (CA) [30] incorporates coordinate information into attention maps, improving the capture of global context and spatial relationships. Shuffling Attention (SA) [31] further divides features into groups for separate spatial weighting, improving performance at the cost of increased computation. Recent efforts have focused on lightweight and efficient attention designs. The Normalization-based Attention Module (NAM) [32] employs weight normalization to suppress irrelevant regions effectively, using only about 30% of the parameters of CAM, yet delivering comparable performance, thereby proving advantageous for deployment in resource-limited environments. Other approaches include Selective Kernel Networks (SKNet) [33], which adaptively adjusts receptive field sizes via kernel selection, and RFACovN [34], which integrates spatial attention directly into convolution through region-wise weighting to capture finer local details.

In summary, from classical channel and spatial attention to recent coordinate-embedded and normalization-based modules, attention mechanisms continue to play a critical role in enhancing the discriminative power and adaptability of object detection networks.

## 3 Method

**Fig 3:**
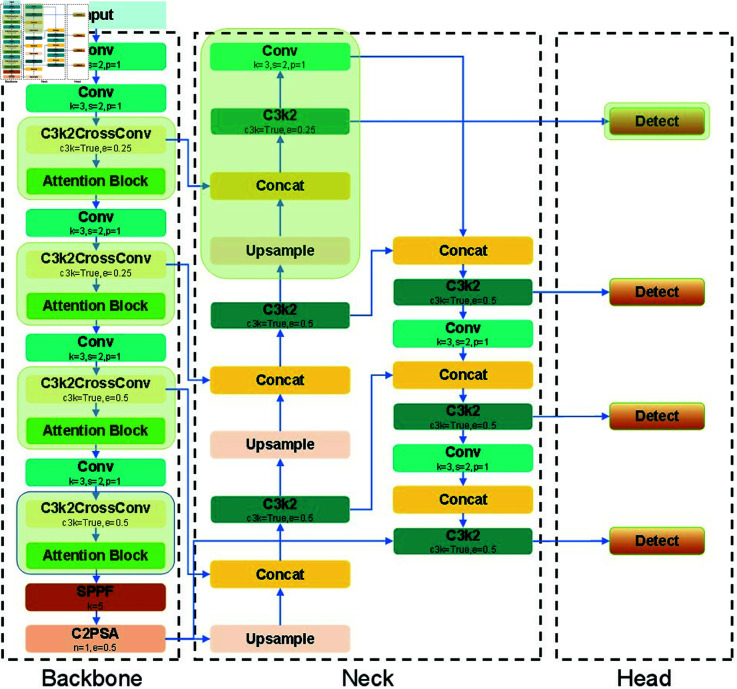
The network architecture of the YOLOv11-ATL model.

This paper proposes the YOLOv11-ATL model based on YOLOv11, which significantly enhances object detection performance. The model introduces a high-precision small-object detection layer that fuses multi-scale features to improve sensitivity to small targets. This layer employs depthwise separable convolution (DWConv), which improves feature extraction efficiency while significantly lowering computational cost, making it particularly effective for detecting small objects in complex backgrounds. To further improve performance in challenging scenes, a global attention mechanism (GAM) is incorporated. GAM captures global contextual information from channel-wise and spatial-wise perspectives, enabling precise attention to important regions while minimizing background noise. Its performance surpasses traditional attention modules such as SE and CBAM. To address the increased computational complexity and maintain real-time applicability, a lightweight C3k2CrossConv module replaces part of the original C3k2 structure. This substitution strikes a balance between accurate detection and efficient processing in terms of speed and resource usage, ensuring the model remains practical without sacrificing performance. In summary, the YOLOv11-ATL model has achieved remarkable improvements in detecting small objects and handling complex scenes.. By comprehensively optimizing the feature extraction, attention mechanism and lightweight design, the model achieves substantial computational efficiency without compromising detection precision, offering robust real-time performance and strong adaptability in resource-constrained environments thus presenting a viable solution for time sensitive object detection tasks. Its network architecture is shown in Fig 3.

### 3.1 Global attention mechanism

The object detection in computer vision frequently encounters intricate visual scenarios, including cluttered backgrounds and irrelevant objects, thereby imposing higher demands on the accuracy and robustness of the model. To address these issues, we apply the Global Attention Mechanism (GAM) [35] to increase the model’s sensitivity to the target area, which significantly enhances detection performance. By incorporating GAM, the model’s sensitivity to the target area is improved, and the reduction of background interference further boosts both its robustness and detection accuracy. Traditional attention mechanisms, such as SENet and Coordinate Attention (CA), perform well in some tasks but have obvious limitations. SENet extracts channel relevance weights through the squeeze-excitation mechanism, but its method ignores spatial dimension information, resulting in insufficient attention to spatial distribution. CA, on the other hand, models the importance of spatial location through location coding, and although it has a better ability to capture local spatial features, it may pay too much attention to the detailed location and lacks the correlation analysis of global information, thus increasing the risk of overfitting. Compared with these methods, GAM employs dual attention mechanisms that operate on both channel and spatial dimensions to comprehensively capture and weight global image features, resulting in significant enhancements in target detection accuracy and robustness. The design of GAM includes both channel attention and spatial attention, with a structure similar to CBAM (Convolutional Block Attention Module), but further optimized for capturing global information. In the channel attention module, a dimension-swapping transformation is applied to the input feature maps, swapping channels (C) and width (W), which improves the model’s ability to capture channel specific features. Then, the channel weights in GAM are calculated through a pair of fully connected layers using ReLU activation functions, followed by a Sigmoid activation to scale the outputs between 0 and 1. Unlike traditional pooling-based spatial attention, GAM’s spatial module employs two standard 7×7 convolutional layers combined with ReLU activation to capture local spatial features more effectively, overcoming the limitations of pooling operations. By modeling features from a global perspective, GAM enables more precise focus on critical regions within the image while suppressing background noise, resulting in a substantial enhancement of the model’s detection performance and reliability in complex environments.

The workflow of GAM can be described through the following steps:

1. **Input Feature Map:** The feature map extracted by the backbone network is input into the GAM module.
2. **Channel Attention Calculation:** First, the channels of the feature map are processed to calculate the importance weights for each channel, and a channel attention map is generated through the MLP.
3. **Spatial Attention Calculation:** Then, spatial positions of the feature map are weighted by using two 7×7 convolution layers, generating a spatial attention map.
4. **Output Weighted Feature Map:** Finally, the weighted and adjusted feature map is passed to the subsequent network for further processing and classification.

**Fig 4:**
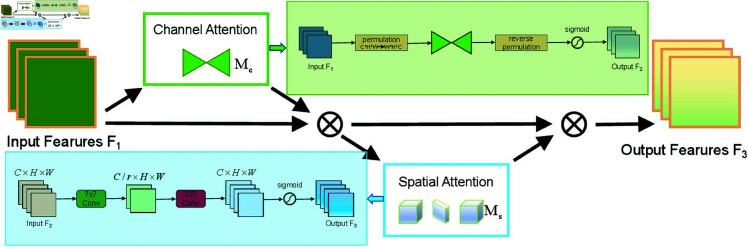
The overall pipeline of GAM.

To better illustrate the workflow and architecture of the Global Attention Mechanism (GAM), Fig 4 shows the overall pipeline of GAM, as defined in Eqs 1 and 2.

$${𝐅}_{2}={𝐌}_{c}({𝐅}_{1})⊗{𝐅}_{1},$$ (1)

$${𝐅}_{3}={𝐌}_{s}({𝐅}_{2})⊗{𝐅}_{2},$$ (2)

Where ${𝐅}_{1}$, ${𝐅}_{2}$, ${𝐅}_{3}$ represent the input layer, intermediate state, and output state, respectively. ${𝐌}_{c}$ denotes the channel attention submodule operation, ${𝐌}_{s}$ represents the spatial attention submodule operation, and ⊗ indicates multiplication.

### 3.2 Tiny object detection layer

Small object detection remains a challenging problem in computer vision, particularly when the background is complex or the targets are very small. Conventional detection methods often face difficulties in accurately recognizing such small objects. To tackle this issue, we introduce a specialized small object detection layer within the neck module of the network, designed to enhance detection accuracy through focused optimization. Using the YOLOv11s model with an input size of 640×640×3 as an example, the neck network first generates a 160×160 feature map with 128 channels via upsampling. This feature map is subsequently fused with the output from the third layer of the backbone network, aligning spatial dimensions and channel numbers. The fused feature map, along with outputs from other detection layers, is then fed into the detection head for classification and localization. This improved architecture markedly strengthens the model’s capability to extract high-resolution features, which is critical for small object detection.

This design is illustrated by the light green regions in the neck and detection head modules of Fig 3, underscoring its significant contribution to overall detection performance. The small object detection layer consists of two core components:

**Feature Pyramid Fusion:** Small objects frequently appear in the lower-resolution areas of feature maps, complicating their detection. Feature Pyramid Networks (FPN) address this by aggregating features across scales. Building on this, our small object detection layer fuses multi-scale feature maps, allowing better preservation and enhancement of small object features from higher-resolution layers.

**High-Resolution Feature Extraction:** Low-resolution feature maps in traditional detectors often lead to loss of small object details. To mitigate this, the small object detection layer incorporates a high-resolution feature extraction module that integrates high-level semantic features with low-level spatial details. This approach helps retain critical small object information, thereby improving detection accuracy and robustness.

### 3.3 Lightweight convolution module

The introduction of the GAM module and the small target detection layer in YOLOv11 has brought about an increase in the number of parameters and computational complexity, although it significantly improves the detection performance of the model. To address this problem and optimize the computational efficiency of the model, this study proposes a method to simplify the YOLOv11 network by reducing the number of model parameters and lowering the computational overhead to optimize the network structure. Specifically, we introduce two lightweight modules, C3k2Ghost and C3k2CrossConv, and comprehensively evaluate their insertion effects at different locations in the network to analyze their impact on model performance and complexity. Under the premise of ensuring that the model performance is not affected as much as possible, this method successfully realizes the lightweight design of the network and effectively improves the computational efficiency of the model.

#### 3.3.1 C3k2 module revisited.

**Fig 5:**
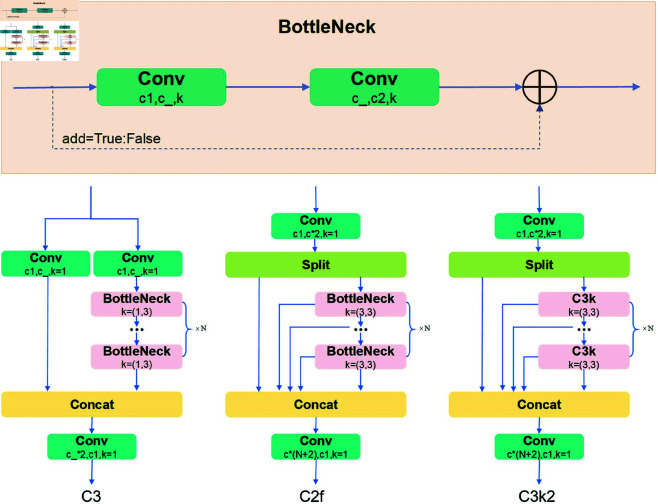
The architecture of the C3, C2f and C3k2 modules.

The architecture of the C3k2 module integrates the characteristics of both the C2f and C3 modules, with the outer layer dominated by the C2f structure and the inner layer employing the C3 structure for feature extraction. In the C3k2 module, when the parameter c3k is set to False, the C3k2 module is equivalent to a standard C2f module. Otherwise, when c3k is set to True, the C3k2 module is based on the C2f structure and replaces the original feature extraction layer (BottleNeck layer) with the C3k layer. The design of the C3k layer is largely consistent with the C3 structure, with key improvements made by adjusting the convolution kernel size from the original BottleNeck to two convolution layers with kernel size 3. This adjustment enhances the feature extraction capability. The specific architecture of the module is shown in Fig 5.

#### 3.3.2 C3k2Ghost module.

Traditional convolution operations rely heavily on a large number of multiply-accumulate operations between input feature maps and convolutional kernels to achieve feature extraction. However, this approach incurs high computational costs, making it less suitable for resource-constrained devices. To address this issue, the GhostConv module introduces a two-step process that decomposes the convolution operation, efficiently generating effective features. First, the GhostConv module employs a standard convolution operation (typically with smaller kernel sizes such as 3×3 or 5×5) to extract "primary features." This step is responsible for capturing the core information of the input feature map, resulting in a small but critical set of feature maps. Next, GhostConv uses a low-cost operation to generate "ghost features." Unlike traditional convolution, this process expands the feature set by applying simple transformations (e.g., 1×1 convolutions or depthwise separable convolutions) to the primary features, avoiding the computational complexity of regular convolutions. Finally, GhostConv combines the primary features produced by the standard convolution with the ghost features generated by the low-cost operation to form a complete output feature map. Through this design of decomposition and recombination, GhostConv effectively expands the receptive field and feature representation capacity of the network while significantly reducing computational overhead. This mechanism allows GhostConv to maintain the model’s expressive power while drastically reducing both the parameter count and computational load, thus significantly improving computational efficiency. Consequently, GhostConv is particularly well-suited for applications in resource-constrained environments, such as embedded systems and mobile platforms, enabling optimized deployment while maintaining model performance.

**Fig 6:**
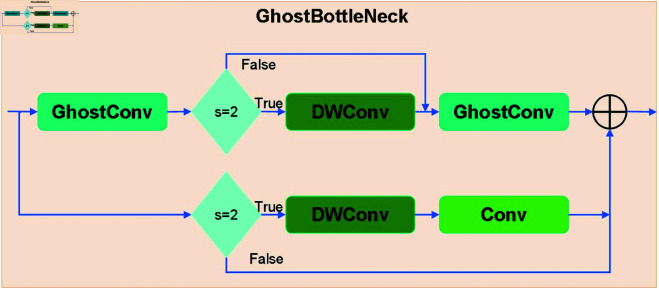
The overall pipeline of the GhostBottleNeck module.

**Fig 7:**
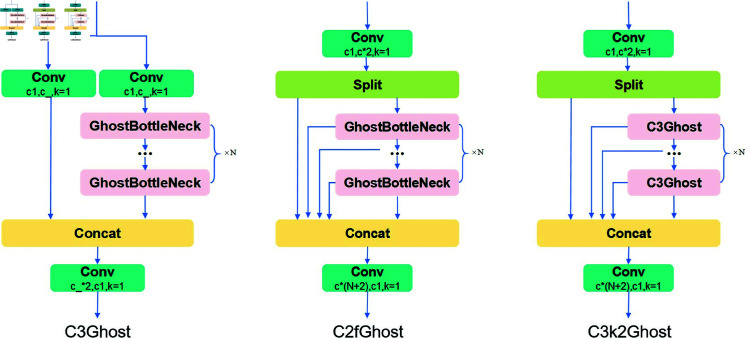
The architecture of the C3k2Ghost module.

The proposed C3k2 module retains the design characteristics of the C2f and C3 modules while enhancing feature extraction capability by incorporating the improved GhostBottleNeck module. Compared to the traditional BottleNeck module, the GhostBottleNeck module integrates GhostConv and Depthwise Separable Convolution (DWConv) and employs a residual structure to improve computational efficiency and feature representation. The GhostBottleNeck module consists of two branches: the main branch and the residual path. In the main branch, a 1×1 GhostConv is first applied to the input feature map for initial feature extraction. When the stride s=2, a 3×3 DWConv module is inserted to downsample or compress the features while enhancing spatial information. This is followed by another 1×1 GhostConv to refine the features and generate the final feature map. In the residual path, when the stride s=2, a 3×3 DWConv processes the input feature map to perform downsampling, which is then followed by a 1×1 standard convolution to match the dimensions of the main branch. When s=1, the input features are directly added element-wise to the features extracted by the main branch without additional downsampling. Finally, the outputs of the main branch and residual path are fused through element-wise addition to produce the final output of the GhostBottleNeck. The detailed architectur of the module is illustrated in Fig 6, Fig 7 demonstrates the integration and optimization of the GhostBottleNeck module within the C3k2Ghost architectur.

#### 3.3.3 C3k2CrossConv module.

**Fig 8:**
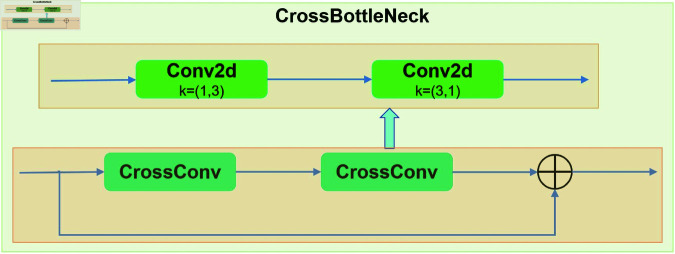
The architecture of the CrossBottleNeck module.

Although the GhostConv operation within the GhostBottleNeck module substantially reduces the computational complexity of the C3k2 module, it inevitably results in the loss of representative information along the channel dimension, thereby adversely affecting the model’s accuracy. To mitigate this limitation, the present study proposes the CrossBottleNeck module, aimed at improving the feature extraction effectiveness. The architecture of the CrossBottleNeck module largely resembles that of the conventional BottleNeck block, with the key distinction being the substitution of the standard convolutional layer by a CrossConv module. Unlike the traditional k×k convolution, which employs a square sliding window kernel, the CrossConv module decomposes the operation into two sequential steps: initially, a 1×k convolution is performed with a horizontal stride of 1 and a vertical stride of *s*; subsequently, a k×1 convolution is applied with a horizontal stride of *s* and a vertical stride of 1. The structural designs of both the CrossConv and CrossBottleNeck modules are depicted in Fig 8. To quantitatively assess the differences in parameter count and computational complexity between the standard convolution and the CrossConv module, this study conducts an evaluation under equivalent input conditions. For an input feature map sized W×W×C, the standard convolution uses a k×k kernel with *C* filters, incorporating padding *p* along the edges. Eqs 3 and 4 provide the corresponding formulations for calculating the floating-point operations (FLOPs) and parameter size of the standard convolution, respectively.

Under equivalent conditions, the CrossConv module was evaluated with an input feature map of dimensions W×W×C. The CrossConv module consists of C dual convolutional kernels, where the first convolution kernel has a size of 1×k, a vertical stride of s, and padding of p along the vertical edges. The second convolution kernel has a size of k×1, a horizontal stride of s, and padding of p along the horizontal edges. Under these conditions, The Eqs 5 and 6 define the floating-point operations (FLOPs) and parameter count of the CrossConv module, respectively.

$${\text{FLOPs}}_{1}=k^{2}C{\left(\frac{W-k+2p}{s}+1\right)}^{2},$$ (3)

$${\text{parameters}}_{1}=k^{2}C,$$ (4)

$${\text{FLOPs}}_{2}=2kC\left(\frac{W-k+2p}{s}+1\right)W,$$ (5)

$${\text{parameters}}_{2}=2kC,$$ (6)

**Fig 9:**
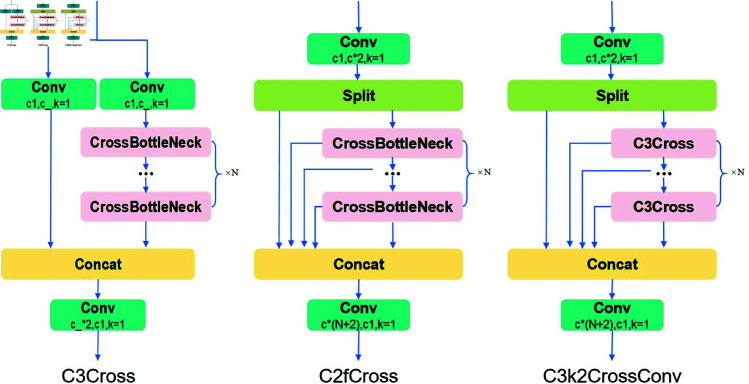
The architecture of the C3k2CrossConv module.

To ensure a sufficiently large receptive field, this study sets the convolution kernel size k to 3 and the stride s to 1. Under these conditions, a comparative analysis of the computational cost and parameter count between standard convolution and CrossConv reveals that standard convolution requires significantly higher computational resources, with its parameter count being approximately 1.5 times that of CrossConv. Similarly, when p=k/2 and s=1, calculations based on the formula show that the computational cost of standard convolution is approximately k/2 times that of CrossConv. Although CrossConv requires two striped convolution operations on a feature map, it achieves more refined feature extraction and generates richer feature information. This improvement not only enhances detection accuracy but also significantly reduces computational demand and parameter count, making it an optimal solution for lightweight model design. The C3k2CrossConv architectur is similar to C3k2, except that the BotteNeck module in C3 and C2f is replaced with a CrossBottleNeck module. The architectur of the C3k2CrossConv module is illustrated in Fig 9.

## 4 Experiment

### 4.1 Experimental environment

To validate the effectiveness of the proposed method, a comprehensive experimental platform was established. The hardware setup consists of an Intel Core i7-11800H processor, an NVIDIA Tesla V100S GPU with 32 GB of memory, and 256 GB of RAM, providing sufficient computational resources for model training and evaluation. The software environment includes the Ubuntu 20.04 LTS operating system, Python 3.9 as the programming language, and PyTorch 1.13.1 as the deep learning framework, ensuring stability and efficiency throughout the experiments. This platform provides robust support for a thorough performance assessment of the proposed approach. Detailed configurations are summarized in Table 1.

**Table 1:** Hardware and software configuration of the experimental platform.

Component	Configuration
CPU	Intel Core i7-11800H
GPU	NVIDIA Tesla V100S (32 GB)
RAM	256 GB
Operating System	Ubuntu 20.04 LTS
Programming Language	Python 3.9
Deep Learning Framework	PyTorch 1.13.1

To ensure the reliability and reproducibility of the experimental results, consistent hyperparameter settings were maintained throughout the entire training process. The specific hyperparameter configurations are presented in Table 2.

**Table 2:** Hyperparametric configuration.

Hyperparameters	Value
Image Size	640 × 640
Batch Size	64
Epoch	300
Optimizer	SGD
Learning Rate	0.01
Momentum	0.937
Weight Decay	0.0005
Warmup Epoch	3
Warmup Momentum	0.8

### 4.2 Dataset

In this study, we utilize the publicly available RDD2022 dataset, which encompasses road damage data from six countries: China, Japan, the Czech Republic, Norway, the United States, and India. The dataset comprises 47,420 images annotated with XML labels, providing comprehensive support for classification, detection, and semantic segmentation tasks. Considering computational constraints and the sufficiency of the 4,878 images within the China subset for our experimental purposes, we selected this subset for model validation. The China dataset is subdivided into two parts: China_M and China_D. The China_M subset includes images captured via a smartphone mounted on a motorcycle, consisting of 1,977 training images and 500 testing images, annotated with 4,650 and 1,046 labels, respectively. The China_D subset comprises drone-captured images, annotated with five damage categories: potholes (D40), grid cracks (D20), repairs (Repair), transverse cracks (D10), and longitudinal cracks (D00). The distribution of these labels is detailed in Table 3. Additionally, the remaining datasets are split into training and validation sets containing 3,410 and 968 images, respectively, with an approximate 7:2:1 ratio across training, validation, and test partitions.

**Table 3:** Types and quantities of defects in the dataset.

Pavement Distress	Distress Class	Quantity
Potholes	D40	255
Grid cracks	D20	756
Road repair	Repair	821
Transverse cracks	D10	1895
Longitudinal cracks	D00	3270

China, as a vast country, exhibits diverse urban road types and conditions, including highways, arterial roads, and secondary urban streets, each subject to distinct traffic patterns, climatic influences, construction practices, and maintenance regimes. These factors contribute to heterogeneous road damage mechanisms, resulting in varied damage manifestations across regions. Consequently, some variability and potential labeling discrepancies may arise within the dataset. Nonetheless, the China subset of RDD2022 provides a comprehensive representation of typical urban road scenarios and damage types, ensuring the evaluation reflects the model’s robustness and generalization capability under realistic, complex conditions. Leveraging the RDD2022 dataset as a benchmark, this study introduces and validates the YOLO-ATL model, integrating novel components such as the Tiny Object Detection Layer and Global Attention Mechanism to significantly enhance detection accuracy for small-scale and complex road damages. Extensive experiments demonstrate that YOLO-ATL surpasses existing baseline methods in mean average precision (mAP) while maintaining an efficient balance between computational cost and detection performance. These results substantiate the model’s suitability as a practical, scalable solution for automated road damage monitoring, with promising applications in intelligent transportation systems and urban infrastructure management.

### 4.3 Performance evaluation metrics

In this study, a set of commonly used evaluation metrics was employed to comprehensively assess the detection performance and computational complexity of the model. For detection performance, Recall, Precision, Mean Average Precision (mAP), and F1 score were selected as key indicators. The model’s predictive capability was primarily evaluated based on false positives (FP), false negatives (FN), true negatives (TN), and true positives (TP) derived from the confusion matrix. Computational complexity was assessed by the number of model parameters and floating-point operations (FLOPs). These metrics collectively provide a thorough evaluation of detection accuracy and resource consumption. The formulas for Recall and Precision are given by:

$$Recall=\frac{TP}{TP+FN},$$ (7)

$$Precision=\frac{TP}{TP+FP},$$ (8)

Given that the RD2022 dataset contains four classes with imbalanced distribution, average Precision and Recall across all classes, denoted as ${\text{Precision}}_{\text{all}}$ and ${\text{Recall}}_{\text{all}}$, are used for evaluation:

$${\text{Precision}}_{\text{all}}=\frac{1}{n}\sum_{i=1}^{n}{\text{Precision}}_{i},$$ (9)

$${\text{Recall}}_{\text{all}}=\frac{1}{n}\sum_{i=1}^{n}{\text{Recall}}_{i},$$ (10)

where *n* is the number of classes and *i* is the class index.

Since there is typically a trade-off between Precision and Recall, relying on a single metric may not fully reflect model performance. Therefore, the F1 score, which balances both, is used:

$$F1\;Score=\frac{2\times {\text{Precision}}_{\text{all}}\times {\text{Recall}}_{\text{all}}}{{\text{Precision}}_{\text{all}}+{\text{Recall}}_{\text{all}}}.$$ (11)

Intersection over Union (IOU) is an essential metric to evaluate the accuracy of predicted bounding boxes by measuring the overlap between predicted ($B_{\text{pred}}$) and ground truth ($B_{\text{gt}}$) boxes:

$$IOU=\frac{B_{\text{pred}}\cap B_{\text{gt}}}{B_{\text{pred}}\cup B_{\text{gt}}}.$$ (12)

Average Precision (AP) assesses the detection accuracy for each category by calculating the area under the Precision-Recall curve:

$$AP=\int_{0}^{1}\text{Precision}(\text{Recall})\;d(\text{Recall}).$$ (13)

Mean Average Precision (mAP) summarizes the overall performance across all categories by averaging their AP values. Common metrics include mAP@0.5 and mAP@0.5:0.95, indicating fixed and varying IOU thresholds, respectively:

$$mAP=\frac{1}{n}\sum_{i=1}^{n}{\text{AP}}_{i}.$$ (14)

### 4.4 Evaluation on different attention mechanisms

This experiment aims to investigate the impact of various attention mechanisms on the performance of the YOLOv11s model in road damage detection tasks. To this end, multiple attention modules were integrated after each C3k2 block within the YOLOv11s architecture to enhance its feature representation capability. The study evaluates the effect of these attention mechanisms on detection accuracy and computational overhead. The compared mechanisms include classical methods such as SENet, CBAM, CA, SA, and ECA, as well as more recent approaches like NAM, GAM, and SKNet. Key evaluation metrics include Recall, Precision, mAP@0.50, mAP@0.50:0.95, F1 score, GFLOPs, and the number of model parameters. The results are summarized in Table 4.

**Table 4:** Detection results of seven attention mechanisms.

Models	Recall_all_	Precision_all_	mAP@0.50	mAP@0.50:0.95	F1 Score	GFLOPs	Parameters (M)
YOLOv11s	86.87%	91.04%	88.2%	76.4%	0.889	21.6	9.43
YOLOv11s+CA	87.32%	91.71%	89.8%	77.3%	0.895	21.9	9.66
YOLOv11s+CBAM	86.55%	91.94%	89.3%	76.9%	0.891	21.9	9.67
YOLOv11s+SENet	86.14%	91.92%	88.8%	76.6%	0.889	21.9	9.67
YOLOv11s+SA	86.41%	92.32%	88.9%	76.6%	0.893	21.8	9.63
YOLOv11s+ECA	87.32%	91.41%	89.5%	77.1%	0.893	21.8	9.63
YOLOv11s+NAM	88.13%	91.81%	89.9%	77.6%	0.899	23.6	10.18
YOLOv11s+GAM	89.17%	92.54%	90.4%	78.2%	0.908	27.4	11.94
YOLOv11s+SKNet	89.78%	95.96%	91.2%	79.1%	0.913	92.5	36.64

As shown in Table 4, incorporating attention mechanisms generally improves the detection performance of the YOLOv11s model. The baseline model (without any attention module) achieves an mAP@0.50 of 88.2% and an mAP@0.50:0.95 of 76.4%, with an F1 score of 0.889, GFLOPs of 21.6, and 9.43 million parameters, demonstrating a lightweight design and solid baseline performance. Among traditional attention modules, Coordinate Attention (CA)improves mAP@0.50 by approximately 1.6% and mAP@0.50:0.95 by 0.9%, with only a slight increase in computational cost. CBAM (Channel + Spatial Attention) achieves a balanced performance gain (+1.1% in mAP@0.50, +0.5% in mAP@0.50:0.95) while maintaining controlled model complexity. ECA and Shuffling Attention (SA) also demonstrate good trade-offs between accuracy and efficiency, suitable for lightweight deployment scenarios. Notably, the NAM enhances mAP@0.50 to 89.9% and mAP@0.50:0.95 to 77.6%, while only moderately increasing the computational cost (GFLOPs of 23.6 and 10.18 million parameters). NAM effectively suppresses redundant regions and achieves significant performance gains under lightweight conditions, making it a strong candidate for resource-constrained applications.

In comparison, the Global Attention Mechanism (GAM) delivers more substantial improvements (2.2% increase in mAP@0.50 and 1.8% in mAP@0.50:0.95), albeit with higher computational demands, suitable for scenarios requiring higher precision. The SKNet achieves the highest detection performance (mAP@0.50 of 91.2%, mAP@0.50:0.95 of 79.1%, and F1 score of 0.913), but its computational cost and parameter count (92.5 GFLOPs and 36.64 million parameters) limit its applicability in real-time or embedded systems.

In summary, all evaluated attention mechanisms contribute positively to enhancing YOLOv11s detection performance. NAM strikes an excellent balance between accuracy improvement and computational efficiency, making it highly suitable for lightweight deployments. Meanwhile, GAM and SKNet, despite higher overheads, represent the upper bound in performance enhancement, suitable for applications with stringent accuracy requirements. This experiment confirms the critical role of attention mechanisms in optimizing YOLOv11s and provides guidance for model selection based on different deployment needs.

### 4.5 Evaluation on tiny object detection layer

Accurate detection of small objects in long-range or low-resolution imagery presents a persistent and critical difficulty within computer vision. Such targets frequently appear blurred or indistinct due to limited spatial resolution, which complicates their differentiation from background elements. This challenge primarily arises from two interrelated issues: (1) the insufficient pixel resolution of small objects on feature maps leads to a substantial loss of critical discriminative information, and (2) the presence of complex background textures further suppresses object-relevant features, increasing the incidence of false positives and missed detections.

To effectively address these challenges, we propose a specialized Tiny Object Detection Layer, specifically designed to improve the representation and detection of small-scale targets. This module leverages fine-grained feature extraction techniques and multi-level feature fusion strategies to enhance the model’s capability in both localization and classification of small objects.

The proposed module was evaluated on a representative benchmark dataset, using two model configurations: the baseline YOLOv11s and the enhanced YOLOv11s-tiny, the latter incorporating the Tiny Object Detection Layer. The experimental results are shown in Table 5.

**Table 5:** Detection performance comparison between YOLOv11s and YOLOv11s-tiny.

Model	Recall_all_	Precision_all_	mAP@0.50	mAP@0.50:0.95	F1 Score	GFLOPs	Parameters (M)
YOLOv11s	86.87%	91.04%	88.2%	76.4%	0.889	21.6	9.43
YOLOv11s-tiny	87.27%	92.84%	89.7%	77.8%	0.900	25.9	9.67

As reported in Table 5, the YOLOv11s-tiny model outperforms the baseline YOLOv11s across all evaluated metrics. Specifically, the mAP@0.50 improves from 88.2% to 89.7%, representing a 1.5% increase, while mAP@0.50:0.95 rises from 76.4% to 77.8%, a gain of 1.4%. Additionally, recall and precision increase by 0.4% and 1.8%, respectively. The F1 score also improves from 0.889 to 0.900, indicating a more balanced and reliable detection capability.

Notably, these performance gains are achieved with only minimal additional computational cost. The GFLOPs of YOLOv11s-tiny increase modestly from 21.6 to 25.9, and the parameter count grows slightly from 9.43 million to 9.67 million. This confirms that the proposed Tiny Object Detection Layer maintains the lightweight and efficient nature of the original model, rendering it suitable for real-time detection in resource-constrained environments.

In conclusion, the integration of the Tiny Object Detection Layer markedly improves the model’s precision in detecting small objects without compromising computational efficiency. This improvement underscores the practical utility of the YOLOv11s-tiny model in scenarios where both precision and real-time performance are required.

### 4.6 Evaluation on lightweight convolution modules

To evaluate the impact of the C3k2Ghost and C3k2CrossConv modules on the lightweight design and detection performance of the YOLOv11s model, this study designs four model configurations as follows: (1) Replacing the C3k2 module in the Backbone of YOLOv11s with the C3k2Ghost module to construct the YOLOv11s-G1 model; (2) Replacing all C3k2 modules in the entire YOLOv11s network with C3k2Ghost modules to construct the YOLOv11s-G2 model; (3) Replacing the C3k2 module in the Backbone of YOLOv11s with the C3k2CrossConv module to construct the YOLOv11s-CC1 model; (4) Replacing all C3k2 modules in the entire YOLOv11s network with C3k2CrossConv modules to construct the YOLOv11s-CC2 model. The experiments evaluate the detection performance and model complexity of these configurations. The findings of the experiment are displayed in Table 6. The YOLOv11s-G1 and YOLOv11s-G2 models, which incorporate the C3k2Ghost module, show significant advantages in terms of complexity. The YOLOv11s-G1 model reduces GFLOPs and parameter count by 8.3% and 7.96%, respectively, but mAP@0.50 decrease by 0.79% and mAP@0.50:0.95 decrease by 0.91%. Further replacing all C3k2 modules in the network with C3k2Ghost modules (YOLOv11s-G2) reduces GFLOPs and parameter count by 16.2% and 17.3%, respectively, but the performance drops more significantly, with mAP@0.50 decreasing by 1.25% and mAP@0.50:0.95 decreasing by 2.75%. This indicates that while the C3k2Ghost module effectively reduces model complexity, its feature extraction capability is somewhat weakened. In contrast, the C3k2CrossConv module strikes a superior balance between efficiency and complexity. The YOLOv11s-CC1 model reduces GFLOPs and parameter count by 6.5% and 6.6%, respectively, while mAP@0.50 increase by 0.45% and mAP@0.50:0.95 increase by 0.39%, outperforming the baseline model. The YOLOv11s-CC2 model further reduces complexity (GFLOPs and parameter count decrease to 19.0 and 8.12M, respectively), and although there is a slight drop in performance, the mAP metrics remain close to the baseline.

**Table 6:** Detection results of lightweight convolution modules.

Models	Recall_all_	Precision_all_	mAP@0.50	mAP@0.50:0.95	F1 Score	GFLOPs	Parameters (M)
YOLOv11s	86.87%	91.04%	88.2%	76.4%	0.889	21.6	9.43
YOLOv11s-G1	85.46%	90.21%	87.5%	75.7%	0.878	19.8	8.68
YOLOv11s-G2	85.55%	89.76%	87.1%	74.3%	0.876	18.1	7.80
YOLOv11s-CC1	86.98%	91.87%	88.6%	76.7%	0.894	20.2	8.81
YOLOv11s-CC2	86.54%	90.76%	87.9%	75.4%	0.886	19.0	8.12

The exceptional results of the C3k2CrossConv module stem from its innovative CrossConv design, which combines two directional convolutions (1×k and k×1) to effectively capture directional features while significantly reducing computational overhead. Additionally, the module retains the residual structure, enhancing feature representation and preserving the flow of information to mitigate the performance decline associated with the feature compression in the C3k2Ghost module. Therefore, the C3k2CrossConv module enhances efficiency while maximizing detection performance, making it an optimal choice for lightweight model design, especially as demonstrated in the YOLOv11s-CC1 model, where it achieves the best balance between performance and efficiency.

### 4.7 Evaluation on YOLO-ATL models

We integrated the GAM, Tiny Detection Layer, and C3k2CrossConv modules into the YOLOv11 network structure and proposed the improved YOLO-ATL model. The ablation experiments verify the contribution of each module in the YOLO-ATL model to performance and complexity. By gradually introducing different modules (GAM, Tiny Detection Layer, and C3k2CrossConv) and analyzing their combinations, the contributions of each module to model performance and complexity were evaluated. Specifically, Tiny represents the addition of the Tiny Object Detection Layer module, which is designed to optimize the detection performance for small objects. CC1 replaces the C3k2 module in the backbone network with the C3k2CrossConv module, reducing complexity and optimizing feature extraction capabilities, and GAM applies to enhance the extraction of key features and improve network robustness. The experimental results, as shown in Table 7, provide a detailed demonstration of the optimization effects of each module individually and in combination on model performance and complexity. These results validate the effectiveness of the collaborative optimization of the modules. The gradual integration of the Tiny Detection Layer, GAM, and C3k2CrossConv modules demonstrates significant optimization effects on the performance and complexity of the YOLOv11s model. When these three modules are combined to form the YOLOv11s-ATL model, overall performance achieves comprehensive improvement. The mAP@0.50 and mAP@0.50:0.95 of YOLOv11s-ATL reach 91.4% and 79.5%, respectively, representing increases of 3.2% and 3.1% compared to the baseline model. The F1 score also significantly improves to 0.918, indicating clear advantages in detection accuracy and robustness. Meanwhile, although GFLOPs and parameter count increase to 30.3 and 11.56M, respectively, the growth remains within an acceptable range. Overall, the YOLOv11s-ATL model achieves the optimal balance between performance and efficiency by leveraging the collaborative optimization of multiple modules while maintaining reasonable complexity. It exhibits significant advancements in small object detection tasks. The experimental results validate the synergy of the modules and the effectiveness and superiority of the YOLOv11s-ATL model.

**Table 7:** Ablation experiments with the modules.

Tiny	GAM	CC1	F1 Score	mAP@0.50	mAP@0.50:0.95	GFLOPs	Parameters (M)
			0.889	88.2%	76.4%	21.6	9.43
✓			0.90	89.7%	77.8%	25.9	9.67
	✓		0.908	90.4%	78.2%	27.4	11.94
		✓	0.894	88.6%	76.7%	20.2	8.81
✓	✓		0.915	91.2%	79.3%	31.7	12.18
✓		✓	0.902	90.1%	78.0%	24.5	9.05
	✓	✓	0.910	90.6%	78.4%	26.0	11.32
✓	✓	✓	0.914	91.4%	79.5%	30.3	11.56

To verify the generality and effectiveness of the YOLO-ATL model, its core improvement modules (GAM, Tiny Detection Layer, and C3k2CrossConv) were applied to different scale models of YOLOv11 (including YOLOv11n, YOLOv11s, YOLOv11m, YOLOv11l, and YOLOv11x). Corresponding improved models were constructed: YOLOv11n-ATL, YOLOv11s-ATL, YOLOv11m-ATL, YOLOv11l-ATL, and YOLOv11x-ATL. By comparing the performance metrics, GFLOPs, and parameter count, this study systematically analyzed the performance improvements and complexity changes brought by the improved modules at different network scales. The experimental results are presented in Table 8.

**Table 8:** Detection results of YOLOv11 models and YOLO-ATL models.

Models	Recall_all_	Precision_all_	mAP@0.50	mAP@0.50:0.95	F1 Score	GFLOPs	Parameters (M)
YOLOv11n	86.87%	89.61%	86.1%	73.2%	0.882	6.5	2.59
YOLOv11n-ATL	88.47%	90.87%	87.6%	75.4%	0.897	8.1	3.71
YOLOv11s	86.87%	91.04%	88.2%	76.4%	0.889	21.6	9.43
YOLOv11s-ATL	89.94%	92.81%	91.4%	79.5%	0.914	30.3	11.56
YOLOv11m	88.23%	91.74%	89.7%	77.8%	0.90	68.2	20.06
YOLOv11m-ATL	90.17%	92.91%	91.7%	79.6%	0.915	95.4	11.56
YOLOv11l	89.64%	92.32%	90.9%	79.1%	0.910	87.3	25.32
YOLOv11l-ATL	90.32%	93.04%	91.8%	79.8%	0.917	122.1	30.97
YOLOv11x	90.14%	92.96%	91.5%	79.7%	0.915	195.5	56.89
YOLOv11x-ATL	90.74%	93.54%	92.3%	80.3%	0.921	273.9	69.72

Based on the experimental results in Table 8, a comparative analysis of the performance of YOLOv11 and YOLOv11-ATL models across different network scales shows that the YOLO-ATL model achieves significant performance improvements at all scales, while maintaining complexity increases within a reasonable range.

In the lightweight model YOLOv11n, the mAP@0.50 of YOLOv11n-ATL improved by 1.7% and mAP@0.50:0.95 of YOLOv11n-ATL improved by 3.0%, with the F1 score increasing by 1.6%. Meanwhile, the increase in GFLOPs and parameter count was limited to 8.12 and 3.71M, respectively, demonstrating good adaptability in lightweight models. For the standard-scale YOLOv11s model, the mAP@0.50 of YOLOv11s-ATL improved by 3.6% and mAP@0.50:0.95 of YOLOv11s-ATL improved by 4.1%, with the F1 score significantly increasing from 0.889 to 0.914, further validating the effectiveness of the improved modules. For larger-scale models (YOLOv11m, YOLOv11l, and YOLOv11x), the improved YOLOv11-ATL series models achieved mAP@0.50:0.95 improvements of 1.5% to 2.0% across the board. For instance, the mAP@0.50 of YOLOv11x-ATL improved from 91.5% to 92.3% and mAP@0.50:0.95 of YOLOv11x-ATL improved from 79.7% to 80.3%, with the F1 score increasing to 0.921, further solidifying its performance in complex scenarios.

In summary, the YOLO-ATL model, through the introduction of the GAM, Tiny Detection Layer, and C3k2CrossConv modules, demonstrates strong adaptability and generalization. Whether applied to lightweight or large-scale networks, it can effectively enhance detection performance while maintaining complexity growth within a reasonable range. This validates the stability and robustness of YOLO-ATL across different network scales.

To further validate the generalization capability of the proposed method, we conducted performance evaluation on the RDD2022 global dataset. Following the original data split ratios, the dataset was divided into training and testing sets. The model was trained on the designated training subset and subsequently evaluated on the global test set to assess its robustness and adaptability across diverse geographic regions and environmental conditions. Evaluation metrics including Precision, Recall, mAP@0.5, and mAP@0.5:0.95 were employed for comprehensive performance assessment. As presented in Table 9, the model maintained high detection accuracy on the global test set, demonstrating strong generalization ability and confirming the practical applicability of the proposed method in complex real-world scenarios.

**Table 9:** Detection results of YOLO-ATL models on the RDD2022 Dataset.

Models	Recall_all_	Precision_all_	mAP@0.50	mAP@0.50:0.95	F1 Score
YOLOv11n-ATL	87.37%	89.64%	86.2%	74.1%	0.885
YOLOv11s-ATL	88.71%	91.56%	90.1%	78.3%	0.901
YOLOv11m-ATL	89.23%	92.01%	90.9%	78.8%	0.906
YOLOv11l-ATL	89.43%	92.56%	91.1%	79.1%	0.910
YOLOv11x-ATL	89.92%	92.74%	91.7%	79.7%	0.913

### 4.8 Evaluation on different models

To comprehensively evaluate the performance of the improved model, this study conducted a systematic comparison with various existing road damage detection algorithms. The comparison models include traditional two-stage anchor-based methods and several one-stage anchor-based and anchor-free methods. Among the two-stage methods, the Faster R-CNN-based road damage detection model proposed by Wang [36] was selected as a representative. The one-stage anchor-based methods include the YOLOv5 model, the SSD model, the EfficientDet model, the YOLOv3-based model proposed by Wang [37], the improved YOLOv7 model proposed by Huang [38], and the BL-YOLOv8 model proposed by Wang [25]. Additionally, recent one-stage anchor-free methods were included, such as the YOLOv6-based model proposed by Yusof [39] and the YOLOX-based model proposed by Li [40]. All experiments were conducted on the same RDD2022 dataset under identical experimental conditions to ensure fairness and reliability in the comparison. This study thoroughly analyzed the detection performance and complexity of these models, validating the advantages of the improved model in terms of accuracy, robustness, and detection efficiency.

Based on the experimental results in Table 10, a comparative analysis of various object detection models reveals that the improved YOLOv11s-ATL model demonstrates significant advantages across multiple performance metrics. Building upon the baseline YOLOv11s model, YOLOv11s-ATL achieves mAP@0.50 improvements from 88.2% to 91.4% and mAP@0.50:0.95 improvements from 76.4% to 79.5%, representing increases of 3.6% and 4.1%, respectively. The F1 score also rises from 0.889 to 0.914. While GFLOPs increase by 40.3% (from 21.6 to 30.3) and parameter count increases by 22.6% (from 9.43M to 11.56M), the performance gains significantly outweigh the increases in complexity. Compared to other models, YOLOv11s-ATL demonstrates superior performance across mAP metrics compared to other models. Specifically, it achieves a 0.7% increase in mAP@0.50 and a 0.6% improvement in mAP@0.50:0.95 over YOLOv8. Additionally, YOLOv11s-ATL outperforms YOLOv5s and YOLOX, with mAP@0.50 improvements of 7.3% and 2.7%, respectively. Further analysis indicates that YOLOv11s-ATL not only excels in detection accuracy but also achieves higher detection efficiency and robustness while maintaining manageable model complexity. Compared to YOLOv7 and YOLOv6, YOLOv11s-ATL shows mAP@0.50 and mAP@0.50:0.95 improvements exceeding 5%, highlighting its exceptional detection capabilities.

**Table 10:** Comparison of performance of different models.

Models	Recall_all_	Precision_all_	mAP@0.50	mAP@0.50:0.95	F1 Score	GFLOPs	Parameters (M)
Faster R-CNN [36]	67.41%	73.27%	73.2%	61.5%	0.702	941.0	28.31
YOLOv5s	86.23%	89.31%	85.2%	73.6%	0.877	15.8	7.02
SSD	68.31%	74.46%	74.6%	63.4%	0.713	62.7	26.28
EfficientDet-D0	61.39%	70.67%	65.4%	58.7%	0.659	5.2	3.87
YOLOv3 [37]	82.72%	85.45%	82.3%	73.4%	0.841	13.0	8.68
YOLOv7 [38]	86.67%	89.84%	85.8%	75.4%	0.882	13.1	6.02
BL-YOLOv8 [25]	89.32%	91.81%	90.7%	78.9%	0.905	25.5	7.82
YOLOv6 [39]	86.74%	89.32%	85.5%	74.9%	0.880	44.0	16.29
YOLOX [40]	87.97%	91.01%	89.0%	77.2%	0.895	15.4	5.06
YOLOv11s	86.87%	91.04%	88.2%	76.4%	0.889	21.6	9.43
YOLOv11s-ATL	89.94%	92.81%	91.4%	79.5%	0.914	30.3	11.56

In summary, YOLOv11s-ATL outperforms most mainstream models in terms of detection accuracy, missed detection rate, and false detection rate, while achieving a reasonable balance between model complexity and computational efficiency. This demonstrates its significant advancements and practical applicability.

### 4.9 Visualization results

**Fig 10:**
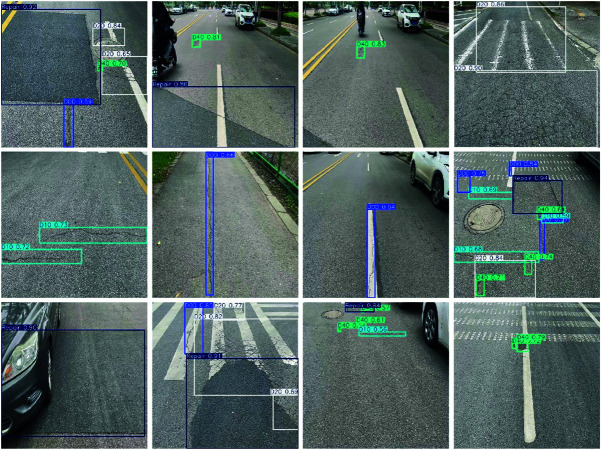
Visualization results of our approach.

To validate the effectiveness of our improved YOLOv11 model in practical applications, we performed a detailed visualization analysis of the detection results. As shown in Fig 10, the model accurately identifies and annotates various types of road damage, including cracks, potholes, and repair zones, even under complex road conditions, demonstrating its robust performance in multi-class damage detection tasks. Furthermore, the model excels in the classification of damage types, effectively distinguishing between different categories of damage, highlighting its exceptional classification capabilities.

For detecting small and inconspicuous damages, such as minor cracks and surface wear, the model maintains high precision, successfully identifying these small targets while significantly reducing the occurrence of false positives and false negatives. Even in scenarios with varying lighting conditions, the model’s detection capabilities remain stable, further proving its adaptability to diverse environmental settings. Notably, the model performs particularly well in detecting small-scale damages, which is crucial for early maintenance and preventing further deterioration of the road.

In summary, the visualization results of these detection tasks fully demonstrate the effectiveness and robustness of our proposed YOLOv11-ATL model in diverse road scenarios. By accurately detecting and classifying various types of road damage while maintaining high precision in small-object detection, the model provides a practical and efficient solution for road infrastructure monitoring and maintenance. Additionally, its outstanding performance underscores its significant potential and value in real-world intelligent transportation systems.

## 5 Conclusions and discussions

This study addresses the inefficiencies, high costs, and susceptibility to human error in traditional road damage detection methods, as well as the limitations of current deep learning approaches in detecting small objects, managing complex backgrounds, and meeting real-time requirements with high computational demands and limited generalization. To overcome these challenges, we propose an improved YOLOv11-ATL model that significantly enhances detection accuracy and robustness through the collaborative optimization of multiple modules.

The model incorporates three key innovations: (1) a Tiny Object Detection Layer leveraging high-resolution feature extraction and multi-scale feature fusion to markedly improve small object detection; (2) a Global Attention Mechanism (GAM) that effectively emphasizes critical regions while suppressing background noise; and (3) lightweight convolution modules (C3k2CrossConv and C3k2Ghost) that optimize network architecture by reducing computational complexity and improving inference efficiency. Extensive experiments on the public RDD2022 dataset demonstrate that YOLOv11-ATL surpasses the YOLOv11 baseline by 3.2% and 3.1% in mAP@0.50 and mAP@0.50:0.95 respectively, alongside a significant increase in F1 score. Although GFLOPs and parameter counts slightly increase, these remain within acceptable limits, reflecting a balanced trade-off between improved performance and resource use. The model shows particularly strong capabilities in small object detection and complex scenarios.

Nevertheless, two main limitations remain: while lightweight design reduces computational overhead, it somewhat affects detection accuracy; and the model’s generalization and adaptability to diverse environments require further enhancement. Future work could integrate advanced attention mechanisms and incorporate self-supervised or multimodal learning to boost robustness and versatility.

In summary, the YOLOv11-ATL model effectively improves the accuracy and efficiency of road damage detection through multi-module optimization, achieving notable advances in small object detection and complex background handling. This work offers a robust and practical solution for intelligent transportation systems and urban infrastructure management, providing novel module designs and validated performance. It enriches the technological foundation of road damage detection and offers valuable insights for future research.

Looking ahead, future studies should emphasize the practical application of this model in real-world settings, including integration with road maintenance, traffic management, and smart city infrastructure, to promote engineering deployment and system integration. Simultaneously, developing intelligent detection hardware such as drone monitoring systems, vehicle-mounted cameras, and mobile platforms will enhance real-time capabilities, automation, and coverage. The deep integration of software and hardware will advance intelligent, efficient, and scalable road monitoring and maintenance solutions, addressing urban traffic safety and public service needs while supporting the development of smarter and more sustainable cities.
